# Facial Contouring in Orthognathic Surgery: The Role of Facial Implants

**DOI:** 10.3390/cmtr19010002

**Published:** 2025-12-24

**Authors:** Gabriel Conceição Brito, Márcio de Moraes, Leonardo Faverani, Sergio Olate

**Affiliations:** 1 Division of Oral and Maxillofacial Surgery, Department of Oral Diagnosis, Piracicaba School of Dentistry, State University of Campinas, São Paulo 13414-903, Brazil; gabriel.bucomaxilo@gmail.com (G.C.B.); mmoraes@unicamp.br (M.d.M.); faverani@unicamp.br (L.F.); 2 Center for Research in Morphology and Surgery (CEMyQ), University of La Frontera, Temuco 4780000, Chile

**Keywords:** facial implants, orthognathic surgery, facial esthetic

## Abstract

Orthognathic surgery restores functional balance and facial esthetics in patients with dentofacial deformities. The use of adjunctive facial implants—made from materials such as porous polyethylene, titanium, or polyetheretherketone (PEEK)—has increased to enhance contour and projection, although standardized guidelines for their selection and integration remain scarce. Following PRISMA-ScR guidelines, a systematic search of PubMed, Scopus, Embase, and LILACS identified studies reporting facial implants placed concomitantly with orthognathic surgery. Eligible studies included case reports, case series, observational studies, clinical trials, and reviews involving human patients, without language or date restrictions. Seventeen studies published between 1998 and 2025 met the inclusion criteria, comprising retrospective and prospective designs, case series, and one technical note. Implants were used in the malar, infraorbital, paranasal, chin, mandibular body, and angle regions. Materials included PEEK, porous polyethylene, silicone, hydroxyapatite, polymethylmethacrylate, and titanium. PEEK was mainly used for patient-specific implants, while porous polyethylene was commonly used as stock implants. Follow-up time, outcome reporting, and study design varied widely, reflecting substantial methodological heterogeneity and predominantly observational evidence. As a result, outcomes were primarily reported qualitatively, limiting comparative assessment and long-term inference. Overall, the available literature suggests that alloplastic facial implants may serve as useful adjuncts to orthognathic surgery for contour enhancement, with outcomes influenced by implant design, surgical expertise, fixation, and soft tissue conditions. However, the current evidence base remains limited, underscoring the need for standardized outcome measures, comparative studies, and longer follow-up to better inform clinical decision-making and future research.

## 1. Introduction

Patients presenting with dentofacial deformities are candidates for orthognathic surgery, a procedure primarily intended to reestablish functional balance by improving occlusion, mastication, and speech [1,2,3]. Nevertheless, the esthetic dimension of orthognathic surgery remains undeniable, and comprehensive treatment planning has increasingly focused on optimizing both functional restoration and facial harmony [3,4,5].

Over recent decades, adjunctive procedures to orthognathic surgery have evolved in response to rising esthetic expectations and the continuous pursuit of more refined, individualized outcomes [5,6,7]. These combined approaches are particularly valuable in complex clinical scenarios, such as patients with facial asymmetry or craniofacial syndromes, in whom achieving correction across the three anatomical planes—axial, sagittal, and coronal—poses a significant challenge [8,9,10]. In such contexts, the integration of complementary techniques may reduce the need for more extensive skeletal movements or invasive osteotomies, such as the Le Fort III procedure [11,12].

The objective of facial implants in orthognathic surgery is to achieve a visible and long-lasting increase in facial soft-tissue volume. Soft-tissue response following orthognathic procedures has also demonstrated a degree of unpredictability [13,14], indicating that the esthetic impact of complementary procedures cannot be fully anticipated. Nevertheless, in addition to facial implants, the use of fat grafts and other alloplastic materials has been reported [15], supporting the notion that volume enhancement represents a key step in optimizing the esthetic outcomes of orthognathic treatment.

Facial implants, introduced in the early 1970s, are now well-established for skeletal reconstruction and esthetic contouring [16,17], and their combined use with orthognathic surgery has gained increasing attention [5,12,18]. They are applied in both the midface and lower face to enhance contour and projection of areas such as the malar eminence, paranasal region, orbital rim, chin, and mandibular angle [11,18,19]. However, evidence remains limited regarding standardized assessment, planning methodologies, and clinical rationale within orthognathic surgery frameworks.

Facial implants may be fabricated from various materials—such as porous polyethylene, titanium, and polyetheretherketone (PEEK)—and are available as either prefabricated (stock) or patient-specific implants (PSIs), the latter designed to conform precisely to individual skeletal anatomy through digital planning and additive manufacturing technologies [20,21].

Given the multifactorial complexity of these cases—including indication criteria, implant selection, surgical approach, and postoperative evaluation—there remains a clear need to define evidence-based strategies for integrating facial implants into single-stage orthognathic procedures. Such strategies aim to maximize both functional stability and esthetic refinement.

Accordingly, the present scoping review aims to map and synthesize the existing evidence regarding the use of facial implants in patients undergoing orthognathic surgery, with particular emphasis on indications, implant materials and designs, clinical outcomes, and their functional and esthetic implications.

## 2. Materials and Methods

### 2.1. Study Design

This study was designed and reported in accordance with the Preferred Reporting Items for Systematic Reviews and Meta-Analyses extension for Scoping Reviews (PRISMA-ScR) guidelines [22]. In accordance with PRISMA-ScR recommendations for transparency and methodological rigor, the protocol for this scoping review was registered in the Open Science Framework (OSF) (Registration ID: 10.17605/OSF.IO/4MG27; available at https://osf.io/4mg27 (accessed on 19 November 2025).

### 2.2. Research Question

The following research question guided the review:

What are the types, indications, surgical techniques, and clinical outcomes reported in the literature for facial implants used concomitantly with orthognathic surgery?

This question was structured using the PCC (Population, Concept, Context) framework:

- Population (P): Patients undergoing orthognathic surgery
- Concept (C): Facial implants (custom-made or conventional)
- Context (C): Simultaneous placement during orthognathic surgery.

### 2.3. Eligibility Criteria

Studies were included if they met the following criteria:

- Involved human patients who underwent orthognathic surgery with simultaneous placement of facial implants (in a single surgical procedure)
- Included study designs such as case reports, case series, observational studies, clinical trials, and reviews
- No restriction on publication date or language.

Exclusion criteria included:

- Studies focused exclusively on orthognathic surgery without reporting any details regarding facial implants
- Narrative reviews without primary data
- In vitro or animal studies
- Studies involving syndromic patients (e.g., craniofacial syndromes).

### 2.4. Information Sources and Search Strategy

An electronic literature search was performed across the following databases: PubMed, Scopus, Embase, and LILACS.

The primary search strategy included combinations of Medical Subject Headings (MeSH) and free-text terms. An example of the search query used in PubMed is (“Prostheses and Implants” [MeSH Terms]) AND (“Orthognathic Surgery” [MeSH Terms] OR “Osteotomy, Sagittal Split Ramus” [MeSH Terms] OR “Osteotomy, Le Fort” [MeSH Terms]) adapted for each database platform.

A. Study Selection and Data Extraction Process

All identified records were imported into START (State of the Art through Systematic Review Tool, LaPES-UFSCar) for management and screening.

Two independent reviewers (GCB and SO) conducted the initial screening by title and abstract. Full texts of potentially eligible studies were then retrieved and assessed against the inclusion and exclusion criteria. Disagreements between reviewers were resolved through discussion and, when necessary, by a third reviewer (MM).

The selection process will be illustrated using a PRISMA-ScR flow diagram. This dual-review process was designed to ensure consistency and minimize selection bias.

B. Data Charting and Synthesis

Data extraction was conducted using a standardized Excel spreadsheet. The following variables were collected from each included study:

- Author(s), year of publication, and country
- Study design
- Patient demographics (age, sex)
- Type of facial implant used (e.g., custom-made, stock)
- Purpose and anatomical region of the implant
- Indication for implant placement (esthetic and/or functional)
- Associated orthognathic surgical procedure
- Clinical and esthetic/functional outcomes
- Reported complications
- Main conclusions.

Descriptive data synthesis will be performed in both quantitative and qualitative formats. The findings will be presented using tables, charts, and narrative summaries, aiming to map the current evidence landscape on this topic.

In accordance with scoping review methodology, the Discussion integrates findings from the included studies with relevant external literature to contextualize the results and support clinical interpretation. This additional literature was not intended to expand the evidence base of the review, but rather to aid in the discussion of key themes identified through the scoping process.

C. Risk of Bias and Quality Assessment

In line with the objectives and methodological framework of a scoping review, no formal assessment of methodological quality or risk of bias was conducted, as recommended by the PRISMA-ScR guidelines.

## 3. Results

### 3.1. Included Studies and Data Extraction

**Figure 1:**
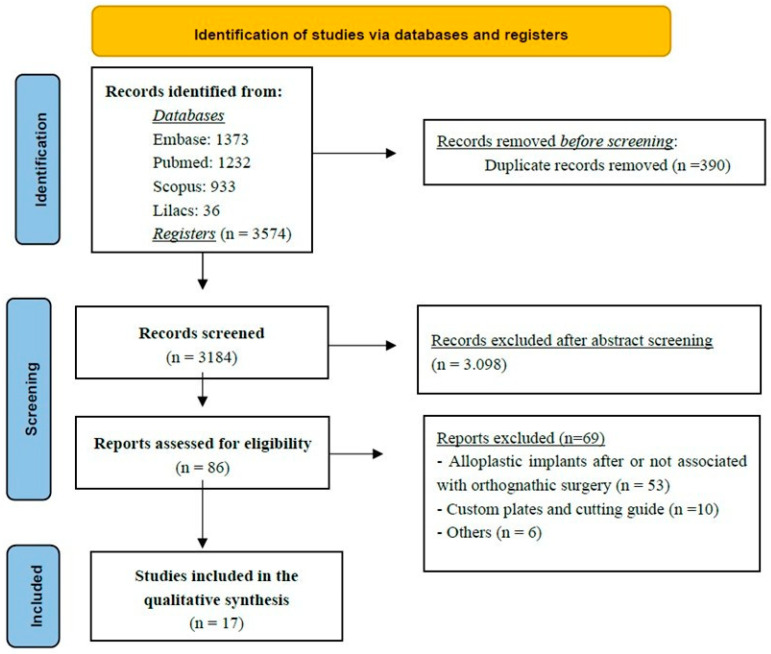
The Flowchart summarizes the study selection process, including identification, screening, eligibility assessment, and final inclusion of studies in the scoping review.

According to the established inclusion criteria, a total of 17 studies were selected for data extraction [7,10,11,18,19,21,23,24,25,26,27,28,29,30,31,32,33]. The overall design and selection process of the included articles are summarized in the flow chart (Figure 1). The studies were published between 1998 and 2025, comprising six retrospective studies [11,20,26,27,32,33], two prospective studies [18,25], seven case series or clinical case reports [7,10,19,21,24,28,29,31], and one technical note [23].

### 3.2. Anatomical Regions and Implant Materials

Among the anatomical regions treated with facial prostheses in conjunction with orthognathic surgery, the included studies reported interventions involving implants designed to modify or enhance the projection of the malar region [11,18,19,26,29,31], infraorbital rim [23], paranasal area [26,27,28], chin (menton) [7,11,19,32], mandibular body [7,10,25,32], and mandibular angle [7,10,11,21,25,26,30,33].

Regarding the materials used for the fabrication of facial implants, the studies described the application of polyetheretherketone (PEEK) [21,26,32], porous polyethylene [11,19,21,25,27,28,29,31], silicone [23], hydroxyapatite granules [18], polymethylmethacrylate (PMMA) [7], and titanium [10,30,33]. PEEK implants were typically associated with patient-specific implants (PSI) manufactured through digital design and additive fabrication technologies, whereas porous polyethylene implants were most often employed as stock implants.

Some studies conducted comparative analyses between different types of facial implant materials, while others focused on the long-term biological behavior and clinical performance of a single material. The key characteristics of each selected study—including the implant material, anatomical region, and notable findings—are summarized in Table 1.

### 3.3. Follow-Up Periods

The follow-up durations varied considerably among the studies and were closely related to their respective study designs. The mean follow-up times (in years) across the studies ranged from 0.5 [25] to 6 [18]. Follow-up protocols were not standardized, showing considerable variability among studies. In most cases, follow-up was limited to the feasible observation period—particularly in case reports and case series—thereby restricting the interpretation of long-term outcomes.

## 4. Discussion

The following sections are structured around key themes identified during data charting of the included studies. Where appropriate, additional literature is cited to contextualize and expand upon these findings.

### 4.1. Indications

Facial implants have emerged as a less invasive alternative to extensive skeletal surgeries such as the Le Fort III osteotomy or zygoma osteotomy, which (initially used to correct midfacial deficiencies and improve esthetics), involved high morbidity, extraoral/intraoral approaches, and significant technical challenges [11,12]. With advancements in biomaterials, the use of alloplastic implants has largely replaced these aggressive techniques for enhancing midface projection and correcting infraorbital and zygomatic deficiencies [18,19]. Simultaneous orthognathic surgery combined with patient-specific implant (PSI) placement has been shown to improve facial symmetry and esthetic outcomes [21,26].

**Figure 2:**
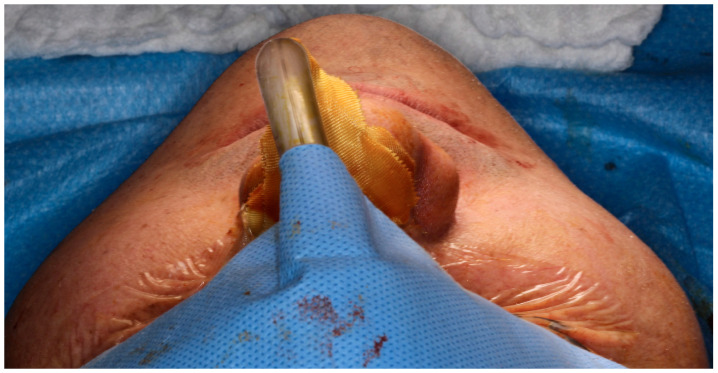
Intraoperative view following Le Fort I osteotomy. A right-side zygomatic implant has been positioned to enhance midfacial projection, while the left side remains without augmentation. The comparison demonstrates an augmentation in zygomatic contour and anterior midfacial projection before bilateral implant placement—Image from the authors’ personal archive.

The midface is key for facial contour, zygomatic projection, and a youthful appearance. Hypoplasia, malar deficiency, and maxillary retrusion disrupt harmony and accentuate infraorbital shadows [18,19]. Correction often involves infraorbital, zygomatic, and paranasal implants to restore malar volume and projection, enhancing facial balance and esthetics [19,28], as demonstrated in Figure 2.

In particular, infraorbital rim deficiency is often associated with a concave facial profile, Class III malocclusion, a shortened upper lip, and prominent nasolabial folds [23]. Additional features such as scleral show, prominent lower eyelid fat pads, and altered globe orientation relative to the infraorbital region are also commonly observed, supporting the indication for implant placement [12,23,34].

Several techniques have been proposed to identify the ideal point for malar augmentation, including those described by Mladick [35], Pendergast [36], Terino [37], and other authors [38,39] who employ different anatomic landmarks and facial lines to determine the most prominent zone. Most recently, the Gridplan method stands out due to its systematic and reproducible approach, dividing the midface into defined areas through a framework of perpendicular vertical and horizontal lines [29]. This grid-based analysis allows three-dimensional evaluation of the relationships between the zygomatic arch, paranasal area, and infraorbital rim, and can be applied both in isolated implant procedures and in conjunction with orthognathic surgery [19,29].

In recent years, the mandibular jawline has emerged as a key determinant of facial attractiveness and perceived youth. There is growing recognition that jawline balance—particularly in patients with facial asymmetry, reduced ramus height, or gonial angle discrepancies—is crucial to overall facial harmony [10,33,40]. To optimize esthetic outcomes, surgeons increasingly combine orthognathic procedures with targeted mandibular augmentation (chin and/or jaw angle) using alloplastic or PSI designs to harmonize chin projection and mandibular angles in height, width, and lateral projection [10,30,40].

Anatomical and esthetic analyses indicate that the ideal gonial angle averages approximately 130°, with sex-related variations influencing mandibular contour and lower-third facial esthetics. The intergonial width should maintain proportionality to the interzygomatic width, typically at a ratio of about 0.83:1, ensuring transverse facial harmony [41]. Proper alignment of the mandibular angles with key facial landmarks—such as the oral commissure and lip level—further contributes to balanced facial proportions [30,41]. In female patients, the gonial angle tends to measure approximately 142° in the frontal view and 125.5° in the profile view, with its vertical position generally aligning with the stomion or upper lip, reinforcing a softer and more harmonious lower facial contour [30,41,42].

Mandibular angle augmentation can be planned using the triangle area concept, defined by lines through the lower incisor apex and posterior ramus to delineate the target region for contour implants. Vertical ramus augmentation restores facial symmetry, aligns the mandibular plane with the Frankfurt plane, and integrates the symphysis and body [21]. Adjustments to the mandibular angle and body width further harmonize intergonial distance and ensure bilateral symmetry.

### 4.2. Implant Materials

**Figure 3:**
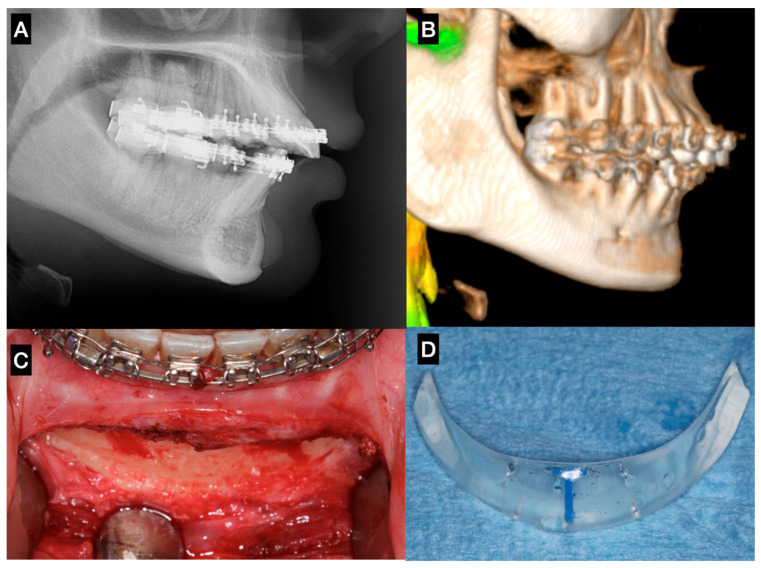
Sagittal chin augmentation using a standard silicone implant in a patient presenting with a Class II skeletal deformity. The patient sought evaluation due to implant mobility detected clinically four years after placement. (**A**) Lateral cephalometric radiograph showing pronounced bone resorption beneath the implant, with proximity to the apices of the mandibular incisors. (**B**) 3D reconstruction confirming a well-defined resorption defect extending toward the mental foramen. (**C**) Post-explantation intraoperative image demonstrating the full extent and depth of the osseous defect. (**D**) Removed standard silicone implant, evidencing its non-anatomic contour and excessive posterior extension, factors that likely contributed to instability and bone resorption—Image from the authors’ personal archive.

A variety of materials have been described for use in facial augmentation and reconstruction procedures associated with orthognathic surgery, particularly in the midface and lower facial thirds [6,12,19]. Fattahi et al. (2017) [23] described the use of silicone implants for infraorbital rim augmentation to correct deficiencies around the infraorbital foramen. Silicone remains popular due to its availability in preformed shapes, ease of placement, and lack of fixation requirements (in some cases). However, its limitations include poor integration, fibrous capsule formation, underlying bone resorption, and a potential for displacement or extrusion over time (Figure 3).

In the study by D’Agostino et al. (2016) [18], porous hydroxyapatite (HA) implants in the midfacial region demonstrated excellent biocompatibility and osteoconductive properties, resulting in stable long-term esthetic outcomes. However, the technique presents certain drawbacks, including the relatively long time required for prosthesis fabrication, a steep learning curve for creating the subperiosteal pocket, and the need for slight overcorrection to compensate for early postoperative volumetric reduction.

High-density porous polyethylene is stable and biocompatible, but it is difficult to adapt due to its rigidity and the need for screw fixation. The reported complication rate is expected to be very low [27]. Nevertheless, the porous structure (100–300 µm) allows fibrovascular and osseous ingrowth, enhancing implant stabilization and resistance to infection—clear advantages over silicone [21]. Other materials have also been described, such as a non-resorbable porous polymeric composite based on polymethylmethacrylate (PMMA) with pore sizes between 150 and 350 µm, as well as silicone, which remains widely used due to its chemical inertness and ease of handling [7].

In the lower facial third, polyetheretherketone (PEEK) and titanium have been extensively studied, particularly in PSI and mandibular augmentation procedures. PEEK offers several advantages, including light weight, radiolucency, and an elastic modulus similar to bone, while not interfering with imaging modalities such as CT or MRI [21,30].

Studies have reported satisfactory esthetic results and comparable symmetry between stock polyethylene implants and customized PEEK implants, with PEEK being more favorable in patient-specific mandibular angle implants [21,26]. However, the lack of osseointegration in PEEK remains a concern among European surgeons, who prefer titanium due to its long-term integration, despite the potential complications associated with osseointegrated materials [43].

Smooth-surfaced titanium implants reduce bacterial adhesion and biofilm formation by 80–90% compared with porous coatings while supporting osteogenic cell compatibility [30]. Patient-specific titanium angle implants further lower displacement risk through precise anatomic fit [10,30]. Titanium also ensures durable outcomes via osseointegration, unlike porous polyethylene, and porous titanium closely matches human bone mechanics, providing structural stability and long-term integration [10,30].

**Figure 4:**
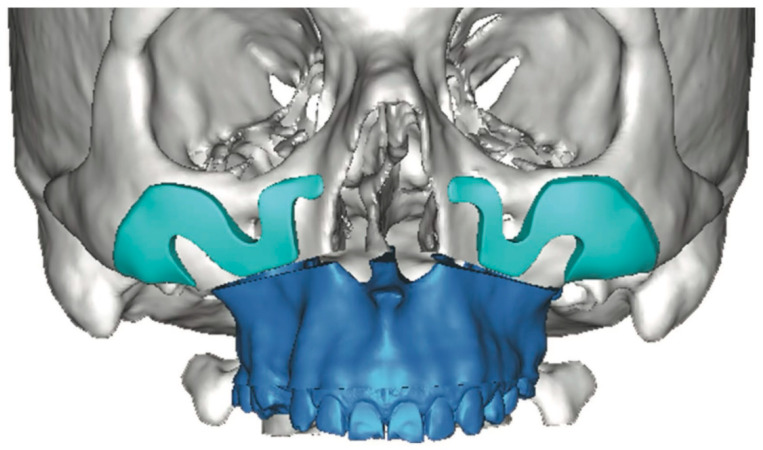
Three-dimensional computer-assisted design illustrates the potential for highly customized implant geometry. However, in this case, the virtual planning lacked adequate anatomical assessment and did not include a rigorous evaluation of the surgical access or the feasibility of implant insertion within the available soft-tissue pocket. These oversights contributed to limitations in the clinical applicability of the proposed design—Image from the authors’ personal archive.

CAD/CAM-designed patient-specific implants (PSIs) enable greater precision and planning, particularly in complex or asymmetric facial cases, offsetting increased cost and planning time by reducing surgical duration and improving esthetic predictability [21,26]. Unlike stock implants, which often require intraoperative trimming and risk asymmetry, PSIs provide faster, more predictable symmetry and volumetric restoration. Screw fixation—usually one or two per implant—ensures stability and prevents postoperative displacement [21]. However, the design of customized implants must account for the practical feasibility of intraoperative positioning and stabilization. If these considerations are overlooked, the implant may not be clinically executable when transitioning from virtual planning to surgical application (Figure 4). Therefore, the surgeon must closely monitor and actively participate in the design process to ensure that the final construct is surgically achievable.

### 4.3. Intraoperative Technical Considerations

Placement of facial implants during orthognathic surgery requires precise planning and delicate technique. Infraorbital rim augmentation after Le Fort I osteotomy with silicone implants is technically demanding, as dissection must expose the infraorbital rim while preserving the neurovascular bundle and periosteal integrity. The implant is contoured to accommodate the infraorbital nerve and positioned flush with the rim to avoid mid-cheek over augmentation. Secure fixation with titanium screws is recommended, particularly when the implant overlaps the Le Fort I plates [11].

**Figure 5:**
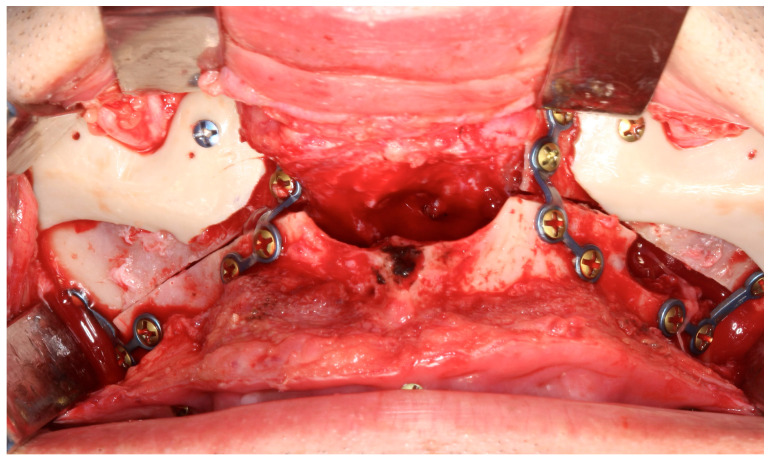
Personalized midfacial implants designed to enhance zygomatic and perinasal projection. Internal fixation was achieved with two screws, and the customized design prevents contact with the titanium plates—Image from the authors’ personal archive.

Placement of malar implants is technically challenging due to the limited surgical field and the need for precise, symmetric bilateral dissection along the zygomatic body and arch. Careful subperiosteal pocket creation is essential to avoid injury to the infraorbital and zygomaticofacial nerves (Figure 5). Slight overcorrection (1 mm) is recommended to account for postoperative tissue adaptation [18,19,28].

Porous polyethylene implants, while promoting tissue integration, require meticulous pocket preparation and a thick soft tissue envelope to avoid erosion or exposure. Excessive handling can damage the implant or surrounding tissues. The introduction of preformed PEEK-PSI has improved intraoperative efficiency by minimizing trimming and manipulation, though precise alignment within the surgical field remains technically demanding [21].

For genioplasty, the intraoral approach is favored to avoid external scarring in patients undergoing concurrent orthognathic procedures [11]. However, this approach restricts visualization and increases soft tissue tension, particularly when inserting large or rigid implants. Dissection must remain strictly subperiosteal to preserve the mental nerves and ensure stable pocket formation [24]. Overextension of the incision or excessive traction may compromise wound closure and elevate infection risk. Proper screw fixation is crucial to counteract dynamic muscular forces during mastication and speech [11,24].

**Figure 6:**
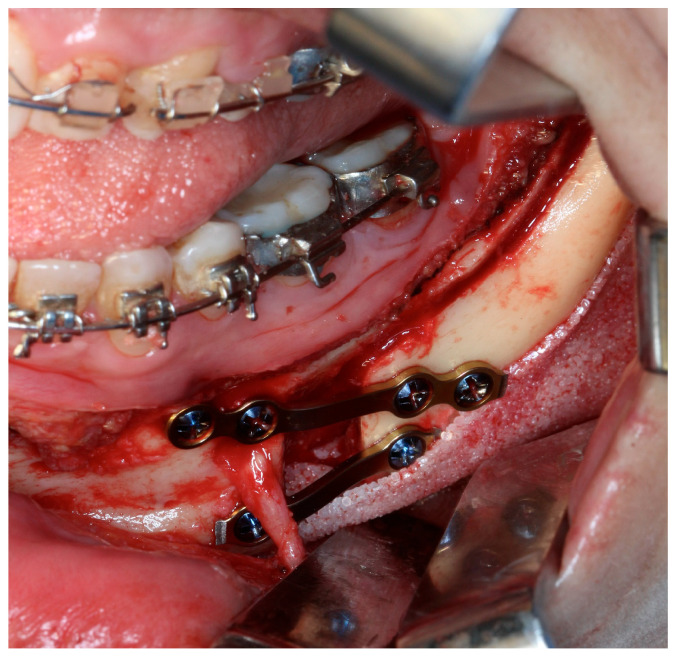
Standard mandibular angle implants. When used in combination with a mandibular osteotomy, adaptation of the implant design is required. Although this remains a viable surgical alternative, it demands additional operative time and meticulous positioning to ensure adequate bone contact and stability. Using PSI, this adaptation can be planned in advance, positioned safely away from the osteotomy line and the fixation point—Image from the authors’ personal archive.

Mandibular angle augmentation during orthognathic surgery is technically demanding (Figure 6) due to the complex anatomy of the pterygomasseteric sling and its functional relationship with the masseter muscle [30]. Careful subperiosteal dissection along the inferior mandibular border is required to preserve the muscular sling, as disruption may cause superior masseter retraction and hollowing of the angle [10,30,33]. Intraoperative strategies to minimize complications include epinephrine infiltration, wide intraoral incisions for visualization, strict subperiosteal dissection, and stable screw fixation. Augmentation should respect the patient’s soft-tissue envelope and masseter strength, particularly in hypodivergent or brachyfacial patients, to avoid over-contouring or implant stress, thereby reducing the risk of malposition, asymmetry, and revision [10,33].

### 4.4. Outcomes

Outcome reporting across the included studies was primarily qualitative, reflecting the absence of standardized outcome measures and the limited availability of comparative data in the current literature. This limitation precludes meaningful quantitative comparison and underscores the exploratory nature of the present scoping review.

Facial implants combined with orthognathic surgery yield predictable and esthetically favorable outcomes when careful technique and sufficient soft tissue coverage are ensured. Porous polyethylene implants perform best under healthy mucoperiosteum, particularly in the paranasal region, with low complication rates. Reported issues, such as excessive prominence or minor asymmetry, may require revision, but proper tissue ingrowth and stable fixation minimize these risks [28].

In a large clinical series, Medpor implants had an overall complication rate of 8.8%, including rare infection, delayed hematoma, transient paresthesia, and mild disproportion, with implant removal required in only 0.7% of cases [19]. Pain and sensory changes were transient, and no long-term migration or allergic reactions occurred. Similarly, Lutz et al. (2020) [11] reported no physical complications over a mean follow-up of 41 months, with an 8% esthetic complication rate that resolved after minor revisions and over 80% patient satisfaction. Malar augmentation performed alongside orthognathic surgery achieved excellent facial symmetry with minimal morbidity.

Patient-specific PEEK implants have emerged as a promising alternative for facial skeletal reconstruction due to their anatomic accuracy, biocompatibility, and intraoperative efficiency. Kerkfeld et al. (2022) [26] reported intraoral exposure in two patients, which was corrected by in situ grinding without further complications. No cases of infection, inflammation, or fracture were noted. Scolozzi et al. (2015) [32] reported no complications in 10 patients with a mean age of 21.3 years who underwent PEEK implants for correction of residual contour chin and/or mandibular defects concomitantly with orthognathic surgery, with a mean follow-up of 1 year.

Titanium patient-specific implants (PSIs) and mesh have shown reliable outcomes when combined with orthognathic surgery for mandibular contour correction. Ramieri et al. (2025) [10] reported stable volumetric augmentation with minimal complications using the Implate system, while Rios et al. (2025) [30] highlighted titanium’s osseointegration and low long-term risk. Stringer and Brown (2009) [33] also confirmed effective mandibular asymmetry correction with angled titanium mesh. Overall, titanium implants—custom or mesh-based—offer predictable, stable results and improved mandibular definition when integrated into the surgical plan.

Despite the potential advantages of simultaneous facial implant placement during orthognathic surgery, several limitations and challenges have been reported. In the acute surgical setting, postoperative edema and ongoing soft tissue adaptation may compromise accurate intraoperative assessment of implant size, contour, and three-dimensional positioning, particularly when stock implants are used [44,45]. This limitation may increase the risk of suboptimal esthetic outcomes, implant malposition, or the need for secondary revision procedures [46]. Consequently, several authors have advocated a staged approach, in which facial implants are placed after completion of orthognathic surgery and stabilization of the soft tissues, allowing for more precise esthetic evaluation, improved surgical planning, and greater predictability of outcomes [20,47,48].

### 4.5. Summary and Future Perspectives

Current evidence suggests that alloplastic facial implants may serve as useful adjuncts to orthognathic surgery, with reported improvements in skeletal contour and soft tissue profile. However, the available data are largely derived from case reports and small observational series, which limits the strength of conclusions regarding safety, effectiveness, and predictability. The growing use of customized, patient-specific implants (PSIs)—particularly those fabricated from PEEK or titanium—appears to offer potential advantages in terms of anatomical fit and surgical planning, although these observations are based on limited clinical evidence. Importantly, reported outcomes remain closely dependent on surgical expertise, appropriate implant fixation, and the condition of the overlying soft tissues. Future studies employing standardized outcome measures, comparative designs, and longer follow-up periods are needed to better evaluate long-term stability, cost-effectiveness, and the impact of digital planning advancements, including more accurate simulation of soft tissue behavior. As the evidence base evolves, these developments may help clarify the role of PSI-based facial augmentation in orthognathic and reconstructive facial surgery.

## Figures and Tables

**Table 1:** Characteristics of the facial implants used and clinical aspects of its application.

Author (Year)	Implant Material	Anatomical Region	Key Distinctive Findings
Marano et al. (2025) [7]	Polymethylmethacrylate	Mandibular angle to the chin	(1) Lower-third volume augmentation with Le Fort I, stable at 1-year follow-up. (2) Following virtual implant design, positive and negative muffle templates were 3D printed using light-curing biocompatible resins
Ramieri et al. (2025) [10]	Titanium (PSI)	Inferior border of the mandible and the posterior margin of the ramus	(1) Simultaneous correction of mandibular advancement, transverse widening, and vertical ramus augmentation. (2) Mean mandibular width gain of 18.1 ± 6.2 mm, with vertical ramus increases of ~6 mm bilaterally.
Rios et al. (2025) [30]	Titanium (PSI)	Mandibular angle implants	(1) BSSO tends to increase intergonial distance → must be considered in implant design. (2) In orthognathic surgery, inserted at the end, after occlusion is established.
Genc et al. (2022) [25]	Porous Polyethylene	Mandibular body and angle implants	(1) BSSO performed first to correct malocclusion. (2) Prefabricated Medpor implants trimmed according to the surgical template. (3) Implants heated in 90 °C saline and molded to fit the mandibular cortex
Kerkfeld et al. (2022) [26]	Polyetheretherketone (PEEK)	Mandibular, maxillary, temporal, zygomatic, periorbital	(1) PEEK implants were virtually planned in a second step (mirroring/superimposition strategy). (2) All patients underwent bimaxillary surgery (Le Fort I + bilateral sagittal split osteotomy), and after the PEEK implant placement
Olate et al. (2021) [21]	Medpor (stock) and PEEK (PSI)	Mandibular angle implants	(1) Stock implants: Triangle shape with ramus and body coverage. size confirmed with a template; implant modified when necessary. Fixed with 1–2 titanium screws. (2) Patient-Specific Implants: Adaptation obtained directly from CAD/CAM (no template needed). Fixed with 1 titanium screw.
Lutz et al. (2020) [11]	High-density porous polyethylene	Malar implants	(1) Simultaneous placement showed no significant difference, but delayed placement was preferred (2) Rationale: gradual facial change, reduced infection risk, short operative time (3) Implants support the lower eyelid in older patients → rejuvenating effect
Findikcioglu el al. (2018) [24]	Porous polyethylene	Genioplasty (chin)	(1) Horizontal sliding osteotomy with 8 mm advancement plus an implant adding 9 mm projection. (2) Mean soft-tissue advancement of 13 mm at the menton region.
Fattahi et al. (2017) [23]	Silicone	Infraorbital rim	(1) It was chosen using sizers (small or medium) for harmony with maxillary osteotomy correction. (2) Implant inserted and positioned as superiorly as possible to augment the entire infraorbital rim length, may rest on Le Fort I fixation plates and secured with titanium screws.
Menezes et al. (2016) [28]	Porous polyethylene	Improvement of the paranasal area	Effect of implants: significant improvement in nasolabial angle and columella inclination in cases of midface hypoplasia
D’Agostino et al. (2016) [18]	Porous HA granules	zygomatic region	(1) Shaping is performed at the end of the Le Fort I orthognathic procedure. (2) Pockets created within the zygomatic bones match implant size (prevent displacement). (3) No internal fixation needed to stabilize implants.
Scolozzi et al. (2015) [32]	polyetheretherketone-PSI	Chin and mandibular defects.	(1) Rapid workflow: Models reviewed, marked, and approved by the surgeon before final manufacturing. (2) Intraoral incision and implants matched bone defect dimensions perfectly; no modifications required 3. Fixation with 1.5 titanium-plate lag screws.
Kwon et al. (2014) [27]	Porous polyethylene	Paranasal	(1) Sub-periosteal reflection performed in the paranasal/piriform area, no need to reflect the anterior nasal spine (2) Thin margins trimmed for anatomical fit; smooth graft transition verified (3) Fixation with 7–9 mm miniscrew
Nocini et al. (2011) [19]	Porous polyethylene	Improvement of the malar area	(1) Clinical parameters for indication: marked mandibular excess, midface hypoplasia, retropositioned upper lip, severe bimaxillary discrepancy, infraorbital shadows (2) Analysis tool: grid-plan midfacial analysis (developed at Verona University).
Stringer et al. (2009) [33]	TiMesh titanium mesh	Mandibular angle	(1) Implant positioning: Medial aspect of mesh tray locked along the medial surface of the posterior border of the ramus and the inferior border of the mandible. (2) Fixation: Only 2 screws required. Minimal forces were applied to the titanium mesh implant.
Nocini et al. (2009) [29]	Porous polyethylene	Improvement of the malar area	(1) Advantages: stability, lower infection risk, fixation with titanium miniscrews, easy to insert and shape (2) Disadvantages: wider intraoral incisions sometimes required (for multiple areas), risk of exposure in thin or scarred skin under tension.
Robiony et al. (1998) [31]	Porous polyethylene	Malar implants	(1) Clinical evaluation and positioning reference by Mladick’s Method (2) Subperiosteal placement via incision from Le Fort I osteotomy and fixed with screws (3) Insertion: anteromedial, anterolateral, or both. The average implant size was 4 ± 0.5 mm.

## Data Availability

The original contributions presented in this study are included in the article. Further inquiries can be directed to the corresponding author.
